# Systematic Review of Literature Examining Bacterial Urinary Tract Infections in Diabetes

**DOI:** 10.1155/2022/3588297

**Published:** 2022-05-17

**Authors:** Santosh Paudel, Preeti P. John, Seyedeh Leila Poorbaghi, Tara M. Randis, Ritwij Kulkarni

**Affiliations:** ^1^Department of Biology, University of Louisiana at Lafayette, Lafayette, LA, USA 70504; ^2^New Iberia Research Center, University of Louisiana at Lafayette, Lafayette, LA, USA 70560; ^3^Department of Pediatrics, University of South Florida, Tampa, FL, USA 33620

## Abstract

This systematic review addresses the central research question, “what is known from the published, peer-reviewed literature about the impact of diabetes on the risk of bacterial urinary tract infections (UTI)?” We examine the results from laboratory studies where researchers have successfully adapted mouse models of diabetes to study the pathophysiology of ascending UTI. These studies have identified molecular and cellular effectors shaping immune defenses against infection of the diabetic urinary tract. In addition, we present evidence from clinical studies that in addition to diabetes, female gender, increased age, and diabetes-associated hyperglycemia, glycosuria, and immune impairment are important risk factors which further increase the risk of UTI in diabetic individuals. Clinical studies also show that the uropathogenic genera causing UTI are largely similar between diabetic and nondiabetic individuals, although diabetes significantly increases risk of UTI by drug-resistant uropathogenic bacteria.

## 1. Introduction

Diabetes mellitus (DM) is a group of chronic metabolic disorders characterized by elevated glucose in blood (hyperglycemia) and urine (glycosuria) resulting either from partial or absolute insulin deficiency due to autoimmune destruction of *β* cells (type 1 DM, T1DM) or from a significant reduction in the ability of cells to respond to insulin (insulin resistance) accompanied by a progressive loss of insulin secretion by *β* cells (type 2 DM, T2DM). Gestational DM (GDM) is marked by insulin resistance during the second or third trimester of pregnancy [1]. According to the National Diabetes Statistics Report, in 2018, an estimated 26.9 million individuals in the US were diagnosed with diabetes, of which 210,000 were children and adolescents younger than 20 years [2]. The global burden of diabetes in 2019 was estimated to be 463 million adults, resulting in 4.2 million deaths [3]. Individuals with poorly regulated diabetes are more susceptible to infections of skin, eye, ear, respiratory tract, gastrointestinal tract, and the urinary tract [4–6]. In 2011, an estimated 10% of total emergency department visits in the US were by diabetic individuals seeking treatment for various infections, of which 30% visits were specifically for urinary tract infection (UTI); in addition, an estimation of 200,000 diabetic individuals required hospitalization for UTI treatment [7]. The overall cost of UTI treatment is 1.2- to 1.5-fold higher for diabetic individuals compared to nondiabetics [4, 8]. The primary objective of this systematic review is to examine clinical studies to elucidate the predictive role of diabetes and associated factors such as hyperglycemia, glycosuria, and immune impairment as well as of sex and age in increasing the risk of UTI in humans. In addition, we also synthesize information from laboratory experiments examining immunological and physiological mechanisms governing the pathophysiology of bacterial ascending UTI in diabetic mouse models.

## 2. Methods

Our central research question was “what is known from the published, peer-reviewed literature about the impact of diabetes on the risk of bacterial UTI?” To address this question, we searched PubMed® (https://pubmed.ncbi.nlm.nih.gov/) on 09/22/2021. Different search terms were used to identify experimental studies using mice and clinical studies as described below. For each search, foreign language articles were removed from the search by automation tool available on PubMed®, while the remaining articles were reviewed by two independent reviewers. The list of included and excluded articles for this review will be made available on request.

**Figure 1:**
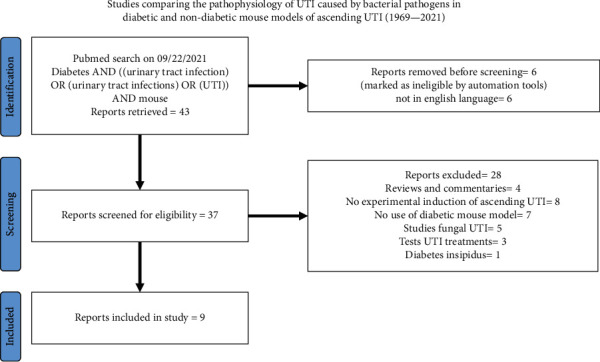
PRISMA flow diagram of describing inclusion and exclusion criteria for experimental studies.

To identify experimental studies to review, we searched PubMed® using terms (Diabetes AND ((urinary tract infection) OR (urinary tract infections) OR (UTI)) AND mouse). We limited our search to articles published between 2000 and 2021.  Figure 1 describes inclusion and exclusion criteria for this search. The main objective of this search was to conduct a comprehensive analysis of differences between diabetic and nondiabetic mouse models in infection parameters such as bacterial organ burden and host immune response following induction of ascending UTI. Hence, we collected data for the following outcomes: strains of mice and uropathogenic bacteria used, animal weight, amount of glucose in blood and urine glucose at the time of induction of ascending UTI, and main findings from the study which are presented in Table 1.

**Figure 2:**
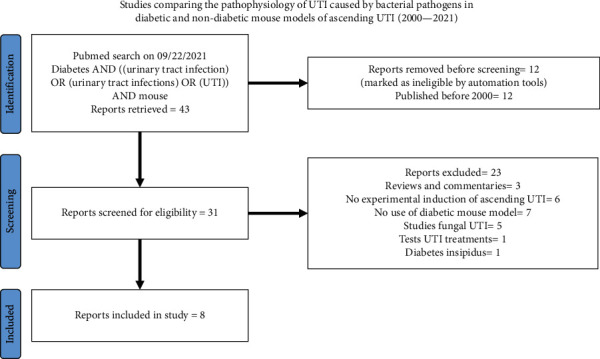
PRISMA flow diagram of describing inclusion and exclusion criteria for clinical studies.

To identify clinical studies to review, we searched PubMed® using terms (Diabetes AND ((urinary tract infection) OR (urinary tract infections) OR (UTI)) AND Epidemiology AND human NOT review). We limited our search to include clinical studies published between 2010 and 2021.  Figure 2 describes the criteria for the exclusion of irrelevant manuscripts and inclusion of manuscripts missed in our search for clinical studies. The main objective of this search was to identify risk factors that increase risk of UTI in diabetic individuals. Hence, we collected the following data: sample size and % women subjects; mean age ± standard deviation (or median age) for diabetic and nondiabetic cohorts; and measures of association such as incidence rate (IR), hazard ratio (HR), incidence rate ratio (IRR), odds ratio (OR), or risk ratio (RR) are presented in Tables 2–5. Where measures of association were not provided, we used published data to calculate them; the calculated ratios are denoted with superscript suffix ^calc^. Any item missing from summary statistics is indicated as NR (not reported); if a study did not recruit nondiabetic individuals, it is indicated as NI (not included).

## 3. Mouse Studies Examining UTI in Diabetes

Over the last decade, the mouse model of streptozotocin (STZ) induced-diabetes has been successfully adapted by UTI researchers to gain insights into the immunology, pathology, and physiology of ascending UTI in diabetes [9–12]. STZ is a DNA alkylating agent administered via intraperitoneal route to destroy a high percentage of endogenous *β*-cells resulting in the reduction in endogenous insulin production, hyperglycemia, and glycosuria; nondiabetic control mice are treated with vehicle (0.1 M sodium citrate, pH 4.5) [13]. Table 1 shows specific STZ dosage and the schedule/duration of STZ treatment used in different studies. Notwithstanding the simplicity and versatility of STZ administration in inducing diabetes in mice in 5-7 days and the immense contribution of this model to our understanding of UTI progression in diabetes, the toxicity, immunosuppression, and lymphopenia induced by STZ are significant confounders when examining the effects of diabetes on urinary immune defenses against uropathogens [14, 15]. These confounders can be avoided by using genetic mouse models of diabetes. Indeed, Murtha et al. have previously induced ascending UTI in two genetic mouse models of diabetes: Lepr*^db/db^* and TallyHo [16]. The monogenic *db/db* mouse model of diabetes is on C57BLKS/J background and carries a spontaneous, autosomal recessive mutation in leptin receptor in the hypothalamus resulting in loss of satiation (hyperphagia), obesity, hyperinsulinemia, hyperglycemia, and glycosuria [17]. Heterozygous (*db*/+) and WT littermates are used as nondiabetic controls. The polygenic TallyHo mouse model of diabetes develops enlargement of islets of Langerhans, hyperglycemia, hyperinsulinemia, hyperlipidemia, and moderate obesity [17]. To experimentally induce ascending UTI, diabetic mice and their nondiabetic controls are inoculated with uropathogenic bacteria via transurethral catheterization. Table 1 summarizes STZ treatment regimen, mouse characteristics such as levels of hyperglycemia and glycosuria, and important results from mouse studies.

The mouse studies reveal that in comparison to the vehicle-treated, nondiabetic controls, diabetic mice were more susceptible to ascending UTI by Gram negative uropathogenic *Escherichia coli* (UPEC) and *Klebsiella pneumoniae* and by Gram positive *Enterococcus faecalis* and *Streptococcus agalactiae* (group B *Streptococcus*, GBS) [9, 12, 16]. However, the post-infection time points at which bacterial burden peaks in the urinary tracts of diabetic mice and the specific nature of the immune response are different for each pathogen. For example, in the bladder and kidneys of STZ-diabetic mice, UPEC and *K. pneumoniae* showed significantly higher bacterial burden at earlier time points (6, 24, and 72 hours post-infection; hpi), while *E. faecalis* showed significantly higher CFU burden much later on days 7 and 14 post-infection [9]. Compared to their nondiabetic counterparts, *db/db* mice showed an increased UPEC burden at 24 hpi in the bladder but not in the kidneys [16]. STZ-diabetic mice also showed an increased GBS burden at 24 and 72 hpi in the bladder but not in the kidneys [12].

The pathogenesis of diabetes is closely associated with significant immune dysregulation. On the one hand, chronic inflammation due to abnormal activation of myeloid lineage cells such as neutrophils and macrophages facilitates diabetes progression, while on the other, diabetes-mediated downregulation of immune defenses increases susceptibility to different infections. Laboratory studies in the context of infections other than UTI have established that DM significantly affects complement activation, neutrophil chemotaxis, phagocytosis, superoxide production, proinflammatory cytokine production, and NETosis, as well as T/B cell activation, production of antibodies, and immunologic memory [18–23]. To define immune defenses protecting diabetic urinary tracts against different uropathogens, the UTI researchers have assayed cytokine levels and immune cell recruitment and have induced experimental UTI in diabetic mice ablated for specific immune effectors [9, 12]. Based on these studies, TLR4-mediated proinflammatory cytokine signaling and neutrophil recruitment appear to be crucial for clearance of Gram negative uropathogens from the diabetic urinary tract: (i) In experiments with *Tlr4*-deficient C3H/HeJ mice treated with STZ to induce diabetes followed by experimental induction of ascending UTI, *Tlr4*-deficient mice show an increased organ burden of UPEC or *K. pneumoniae* in comparison to *Tlr4*-wild type C3H/HeN control mice [9]; (ii) UPEC-infected STZ-diabetic mice show a reduced expression of pro-inflammatory cytokines IL-6, CXCL1 (KC), CXCL2 (MIP2), and CCL2 (MCP-1) and significantly reduced neutrophil infiltration into infected bladder tissue [10]. In addition, UPEC-infected *db/db* diabetic mice also show a suppression of insulin receptor (IR)-PI3/AKT signaling axis in the renal intercalated cells resulting in reduced production of antimicrobial peptides defensins, cathelicidin, non-enzymatic RNases, and lipocalin-2, which may in turn be implicated in higher UPEC burden in infected kidneys [16]. GBS-UTI also induces cathelicidin production, although it may be ineffective in urinary defense against GBS as cathelicidin ablated *camp*^−/−^ mice treated with STZ do not show increased bladder GBS burden compared to their *camp*^+/+^ counterparts [12]. A few clinical studies have also examined urinary immune defenses in diabetics: (i) in a prospective cohort study, T2DM patients (*N* = 197) with *E. coli* UTI had significantly diminished activity via ficolin 3-mediated lectin and alternative pathways of complement activation compared to nondiabetic (*N* = 196) controls; complement activity via classical and mannose-binding lectin pathways was unaffected [24]; (ii) compared to nondiabetic women with asymptomatic bacteriuria (ASB), diabetic women with ASB had lower urine leukocyte count and reduced levels of IL-6 (*P* < 0.001) and IL-8 (*P* = 0.1) [25].

Interestingly, both UPEC and *K. pneumoniae* induce significant renal neutrophilia and inflammation of tubular epithelium in STZ-diabetic mice, which appears to be ineffective in controlling UPEC infection as the renal tubules from UPEC-infected diabetic mice showed large, extracellular biofilm-like communities of UPEC; *K. pneumoniae* infected diabetic mice do not show biofilm-like communities in their renal tubules [9]. In contrast, the mast cell activity in GBS-infected diabetic urinary tract appears to be detrimental to the host: Compared to the vehicle-treated, nondiabetic controls, STZ-diabetic mice show a significantly higher recruitment of bladder mast cells (ckit^+^ and Fc*ε*RI^+^) 24 h after GBS infection; however, treatment of STZ-diabetic mice with mast cell degranulation inhibitor cromolyn sodium significantly reduces organ GBS burden [12]. Lastly, it is noteworthy that in a 1994 report from Japan, researchers observed reduced neutrophil bactericidal activity accompanied by reduced CD4^+^T-helper and B cell types, and increased macrophages infiltrating the urinary bladder mucosa of STZ-diabetic mice transurethrally infected with *E. coli* compared to their nondiabetic counterparts; differences in bacterial organ burden, dissemination, or disease severity between diabetic and nondiabetic mice were not described [26]. In summary, laboratory experiments have revealed that diabetic urinary tracts are more susceptible to infection due to immune deregulation although future research is needed to identify specific molecular and cellular immune defenses playing a consequential role in shaping the pathophysiology of UTI in diabetes.

## 4. The Etiology of Urinary Colonization in Diabetes

**Figure 3:**
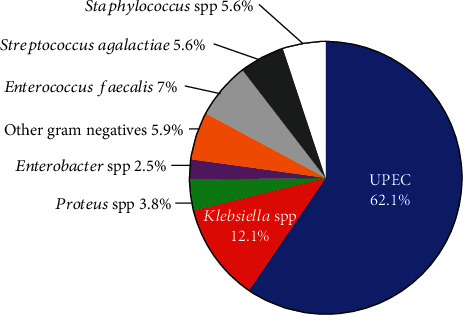
Microbial etiology of UTI in diabetic individuals.

The bacterial pathogens that cause ASB, community-acquired UTI, and healthcare-associated UTI in diabetic individuals are similar. Here, ASB is defined as positive urine culture without UTI symptoms; community-acquired UTI refers to UTI that occurs in community affecting individuals who are not hospitalized or receiving homecare; while healthcare-associated UTI refers to UTI affecting hospitalized patients under peri-/postoperative care, elderly requiring homecare, and/or those with indwelling urinary catheters and ureteric stents. Among Gram negative pathogens, *E. coli* (also known as UPEC) is the principal etiology of ASB and UTI in diabetic individuals followed by *Klebsiella* and *Proteus*, while among Gram positives, *Enterobacter* is the major uropathogen followed by *Staphylococcus* spp. (*Staphylococcus aureus* and coagulase negative staphylococci) and *GBS* [27–36] as shown in Figure 3. Even among nondiabetic individuals, a vast majority of UTI (including both complicated and uncomplicated cases) are caused by Gram negative bacteria such as UPEC (65-80%), followed by *Klebsiella* spp. (3.5-13%), *Pseudomonas aeruginosa*, and *Proteus mirabilis* (2-6%), while the rest are caused by Gram positive bacteria such as *Staphylococcus* spp. (4-6%), *Enterococcus faecalis* (4-7%), and *GBS* (3%) and Candida (1%) [37, 38]. The comparison of percentages of causative agents of UTI between diabetic and nondiabetic individuals shows that DM does not favor urinary colonization by specific bacterial pathogens over others. Indeed, studies from Turkey [39], the Netherlands [40, 41], France [42], and Pakistan [29] have shown that the % distribution of uropathogenic strains recovered from diabetic and nondiabetic individuals is similar, although a few studies have observed positive correlation between presence of DM and increased prevalence of certain uropathogens: (i) On a study of hospitalized patients with UTI (DM = 404; no DM = 959), *Klebsiella* spp. was ~2-fold more common as a uropathogen in diabetic individuals compared to nondiabetics (*P* = 0.011), although there was no significant difference in the number of diabetic and nondiabetic individuals from whom UPEC, *Proteus* spp., *Pseudomonas* spp., and *Enterococcus* spp. were isolated as causative uropathogen [30]; (ii) a study from Argentina reported that ~7% individuals with GBS-UTI were diabetic [43]; (iii) in a prospective study of diabetics with culture positive UTI diagnosis (*N* = 252), compared to subjects with good glycemic control (Hb_A1C_ = 5.4% ± 0.5,*N* = 55), those with poor glycemic control (Hb_A1C_ = 8.3% ± 1.5; *N* = 197) showed a 1.1-fold and 1.25-fold increase in the detection of UPEC and *K. pneumoniae*, respectively [44]; (iv) in a study from a French hospital (DM = 72; no DM = 227), DM increased the odds of polymicrobial (*E. faecalis*, *E. coli*, and *P. aeruginosa*) bacteriuria (OR adjusted for age and sex = 2.0; *P* = 0.04) [45]; and (iv) in pregnant women with pre-gestational DM (DM = 150; no DM = 294), diabetes significantly increased risk of bacteriuria caused by GBS (OR = 2.47) [46]. Whether the DM increases susceptibility to pathogens with specific virulence features is not examined extensively, although in a study, the presence of DM was not correlated with the recovery of hypermucoviscous *K. pneumoniae* from UTI patients [47].

However, it is particularly alarming from the viewpoint of UTI treatment that diabetic individuals are at >2-fold higher risk of UTI by drug-resistant uropathogens. For example, (i) a study from Singapore noted a significant increase in the susceptibility of diabetic individuals to uropathogens from amoxicillin-clavulanate resistant *Enterobacteriaceae* family with adjusted OR (aOR) of 2.54 (*P* = 0.03) [48]; (ii) 85.2% UPEC isolated from diabetic patients with UTI were multidrug resistant (*N* = 1520; mean age = 58 years) in a study from Pakistan [49]; (iii) DM increased susceptibility to UTI by multidrug resistant bacteria (OR = 2.05; *P* = 0.001) in kidney transplant recipients from Brazil [50]; (iv) DM increased risk of UTI caused by extended spectrum cephalosporin-resistant *Enterobacteriaceae* (OR = 2.7, *P* = 0.007) in a study from the US [51]; (v) DM increased the risk of UTI by extended spectrum *β*-lactamase (ESBL) producing *E. coli* and *K. pneumoniae* with OR (adjusted for age and sex) = 5.51 (*P* = 0.036) in a study from the UK [52], with OR = 4.4 (*P* = 0.002) in a study from the US [53], adjusted OR = 3.2 (*P* = 0.051) in a study from Norway [54], and OR^calc^ = 1.96 (*P* = 0.032) in a study from Spain [55]; (vi) diabetes increases the risk of UTI by uropathogens resistant to quinolone antibiotics with OR = 3.5 (*P* < 0.01) in a study from Taiwan [56] and OR = 2.09 (*P* = 0.04) in a study from France and to cephalosporins with an OR = 3.67 (*P* = 0.05) [42]; (vii) in a cohort of patients with *E. coli* UTI (DM = 190; no DM = 81), significantly higher number of diabetics (90%) compared to nondiabetic controls (67.2%) were infected with strains resistant to one or more cephalosporins [57]; and (viii) in renal transplant recipients with *K. pneumoniae* bacteriuria (*N* = 100), DM increased the risk of carbapenem-resistant *Kp* bacteriuria (aOR = 5.5; *P* = 0.01) which is in turn associated with graft failure and mortality [58].

In summary, the species profile of UTI-causing pathogens from diabetic individuals is not different from that in nondiabetics, although diabetes significantly increases the risk of urinary colonization by drug resistant uropathogenic bacteria.

## 5. Diabetes as a Risk Factor of ASB and Symptomatic UTI

Multiple studies show that DM increases the risk for ASB: (i) In a study of elderly individuals in a nursing home in Sweden (*N* = 385, mean age = 87 ± 6.7 years) diabetes increased the risk of ASB (aOR (adjusted for age, sex, and serum vitamin D level) = 2.3; *P* = 0.014) [59]; (ii) in a study from Cameroon (DM = 154, no DM = 111), ASB was 1.5-times more prevalent in diabetics, with *Candida* being the more common etiology of ASB in diabetics [60]; (iii) in a cohort of elderly women (mean age = 71.9 years), diabetes increased the risk of ASB (OR = 2.49; *P* = 0.041) [61]; and (iv) in a cohort of pregnant women from Netherlands (DM = 202, no DM = 272 at 12 weeks of pregnancy, diabetes increased the risk for ASB (RR = 2.02), although by week 32 of pregnancy, diabetic versus nondiabetic RR for ASB was 1.06 [62]. In summary, the clinical studies show that in comparison to their nondiabetic counterparts, individuals with DM are at approximately 1.5–3-fold higher risk of ASB and 2-fold higher risk of community-acquired UTI (Table 2) as well as healthcare-associated UTI (Table 3).

## 6. Diabetes as a Risk Factor of UTI Complications

Multiple studies have shown that diabetes increases the risk for UTI complications such as recurrent UTI, bacteremia, requiring hospitalization for UTI treatment, and 30-day mortality: (i) In elderly individuals with diabetes-associated chronic kidney disease (CKD; N = 79,887; mean age = 59.6 years), the presence of age-associated frailty was a significant risk factor of UTI and urosepsis [63]; (ii) in a study from Greece, diabetic individuals (DM = 19; no DM = 81; median age = 60 years) were more susceptible to recurrent UTI (defined as ≥3 UTI in one year) with an OR of 5.5 (*P* = 0.006) [64]; (iii) in an Australian study (DM = 396; no DM = 2391; overall mean age = 37.1 years), diabetic individuals were more likely to be hospitalized for UTI treatment (OR^calc^ = 2.8; *P* < 0.001) requiring ~1.5 days longer hospital stay (*P* = 0.04) compared to nondiabetics [65]; (iv) in a matched control study from the US (*N* = 179,580; mean age = 56 years), compared to nondiabetics, the UTI recurrence within three months of first diagnosis of T2DM was ~3-times (*P* < 0.0001) more common [66]; (v) in a prospective study of hospitalized diabetic patients with advanced CKD (glomerular filtration rate< 30 ml/min; *N* = 88; mean age = 68 years), individuals with higher fasting glucose showed slower (> 9 days) renal recovery after an episode of UTI and ~ 3 times higher susceptibility to developing Gram negative bacteremia [67]; (vi) in a study of elderly Dutch individuals, compared to nondiabetic controls (*N* = 718, median age = 64 years), diabetic individuals (*N* = 140; median age = 73 years) were at higher risk of recurrent UTI (OR (adjusted for age and cardiovascular disease) = 2.2; *P* = 0.017, higher risk for bacteremia (OR = 1.2; *P* = 0.037), and higher risk of 30-day mortality (OR = 2.0; *P* = 0.007) [40]; (vii) in a retrospective study from Taiwan, diabetic patients with late-stage CKD (glomerular filtration rate ≤ 30 ml/min; *N* = 225; mean age = 63.5 years) had an increased risk of acute kidney injury following an episode of UTI [68]; (viii) in a cohort of Japanese patients requiring hospitalization for the treatment of bacteremia resulting from UTI (*N* = 70; mean age = 68 years), 17.1% were diabetic [69]; (ix) compared to nondiabetic controls (*N* = 81; mean age = 66 years), diabetic patients with *E. coli* UTI (*N* = 190; mean age = 69 years) showed a 1.2-fold higher incidence of urosepsis [57]; (x) in a cohort of elderly hospitalized for UTI treatment (*N* = 251, mean age = 65.3 years), DM significantly increased the risk for death (OR = 22.66; *P* < 0.01) in a 30-day period following hospitalization [70]; and (xi) in a cohort of Dutch women, compared to nondiabetic controls (*N* = 6958; mean age = 51 years), diabetic subjects (*N* = 340; mean age = 65.5 years) were at a higher risk of recurrent UTI (OR (adjusted for age) = 2.0; *P* < 0.001) [71]. In summary, it is evident from clinical data that diabetes significantly increased risk of UTI complications such as recurrent UTI, bacteremia, and for requiring hospitalization for UTI treatment, with an important caveat that in majority of studies examining association between DM and UTI complications, diabetic subjects are ≥60 years of age. Clinical studies do not support that DM increases the risk of ASB progression to symptomatic UTI.

## 7. Epidemiological and Laboratory Studies Examining Hyperglycemia as a Risk Factor of UTI

The loss of *β* cell mass and/or function in both T1DM and T2DM clinically manifests as hyperglycemia, which is a biochemical hallmark of DM [1]. Fasting (8 h without food intake) plasma glucose ≥126 mg/dl or 2 h plasma glucose ≥200 mg/dl during 75 g oral glucose clearance test is diagnosed as diabetes [1]. Generated by non-enzymatic addition of glucose to hemoglobin *β* chain, plasma glycated hemoglobin (Hb_A1C_) >6.5% (>48 mMol/Mol) is assayed to monitor long-term glycemic status [1, 72]. Chronic hyperglycemia results immune dysfunction, microvascular (nephropathy, retinopathy, and neuropathy) and macrovascular (coronary artery disease and stroke) complications, and infections. Given its adverse health effects, glycemic control using a combination of lifestyle changes (diet and physical exercise) and pharmacological agents is of paramount importance for long-term health in diabetic individuals. While discussing whether hyperglycemia affects the risk of UTI, glycemic control is broadly categorized into poor (Hb_A1C_> 8.5%), moderate (Hb_A1C_ = 7—8.5%), and good (Hb_A1C_< 7%), although in individual studies discussed below the criteria for categorizing glycemic control may deviate moderately from these definitions. Clinical studies confirm that poor glycemic control increases the risk of ASB and UTI by ~2-fold. For example, (i) poor glycemic control was a significant risk factor for ASB (OR = 1.97; *P* < 0.001) compared to control with good glycemic control in a study from Pakistan [28]; (ii) in a retrospective study of hospitalized trauma patients from the US, compared to nondiabetic controls (%HbA1C< 5.7), the risk of UTI increased in those with good glycemic control (RR = 1.48; *P* < 0.001) as well as poor glycemic control (RR = 1.83; *P* < 0.001) cohorts [73]; (iii) compared to diabetics with good glycemic control, adjusted OR for UTI was 1.12 (*P* = 0.06) for diabetics with modest glycemic control and 1.18 (*P* = 0.04) for diabetics poor glycemic control [74]; (iv) in a study of T1DM women (*N* = 572; average DM diagnosis = 29.8 ± 5 years), UTI prevalence doubled from 9.8% to 20.2% with increasing glycemia from Hb_A1C_< 7.3% to Hb_A1C_> 8.3%; this study also calculated that every unit (1%) increase in Hb_A1C_ level is associated with 21% increase (*P* = 0.02) in the frequency of UTI in 12 months prior to plasma glucose measurement when adjusted for subjects' race, hysterectomy status, urinary incontinence, sexual activity in the past 12 months, peripheral and autonomic neuropathy, and nephropathy [75]; (v) in a study from Australia, compared to nondiabetic individuals, individuals with blood glucose ≥5.3 mMol/L were more susceptible to UTI (OR = 2.1) [65]; (vi) when stratified for other demographic and clinical data, UTI was more common (hazard ratio, HR = 1.29–1.4) in diabetic individuals with poor glycemic control [76]; and (vii) in a study from the UK, compared to individuals with moderate glycemic control (*N* = 79,974), those with poor glycemic control (*N* = 30,089) showed RR = 1.24 for UTI [77].

In summary, studies have established a positive correlation between increasing hyperglycemia and the risk of UTI albeit with an important caveat that they rely on the measurement of a single baseline Hb_A1C_ value (or fasting blood glucose value), which are then correlated with a future incidence of UTI, days, weeks, or months after plasma glucose measurement. A retrospective case control study (*N* = 510 UTI cases, 2463 control) involving hospitalized patients also shows that short-term changes in plasma glucose do not significantly alter UTI risk [78]; the improvement in glycemic control also does not immediately translate into a corresponding reduction in the risk for UTI as observed in a study of T2DM patients from the US (*N* = 2,737), where ~53% of study participants experienced a 1.5% decrease in mean Hb_A1C_ levels following a switch from oral antidiabetic therapy to insulin, although this did not affect the risk for UTI in one year period after switch (*RR* = 1.04) [79].

In laboratory experiments, uroepithelial cells from diabetic individuals with poor glycemic control showed a higher adherence to type 1 fimbriated *E. coli* [80]. When colonizing urinary bladder, UPEC uses type 1 fimbria to adhere to glycoprotein uroplakin lining the apical surfaces of uroepithelial umbrella cells [81]. In UPEC-infected STZ-diabetic mice, advanced glycation end products (AGE) accumulated on bladder epithelium provide alternative binding receptors for type 1 fimbriae on the surface of UPEC in turn facilitating bladder colonization [11]; however, whether the accumulation of AGE on diabetic uroepithelium facilitates urinary colonization by uropathogenic bacteria in humans is unknown.

In summary, these observations indicate a positive correlation between poor glycemic control and increasing risk of community- and hospital-acquired UTI. Interestingly, poor glycemic control does not appear to be a significant risk factor UTI progression into urosepsis [30, 57].

## 8. Epidemiological and Laboratory Studies Examining Glycosuria as a UTI Risk Factor

The presence of >25 mg/dl glucose in urine (glycosuria) can be a direct manifestation of elevated levels of plasma glucose due to diabetes mellitus [82, 83]. While uropathogenic bacteria exhibit robust growth in nondiabetic urine by switching their metabolism towards amino acid utilization via TCA cycle and gluconeogenesis [84], glycosuria is expected to further enhance growth of uropathogens by facilitating glycolytic metabolism. Indeed, supplementation with 100, 200, or 1000 mg/dl glucose (corresponding to low or severe glycosuria, respectively) enhanced growth of *E. coli* in human urine [9, 83]. Interestingly, the urine from diabetic individuals without glycosuria did not enhance *E. coli* growth, which suggests that glucose is the principal mediator of enhanced bacterial growth observed in glycosuria [83].

Glycosuria can also result from administration of SGLT2i (sodium-glucose co-transporter-2 inhibitors, *aka* gliflozins), oral antidiabetic drugs that induce normoglycemia by preventing reuptake of glucose in the proximal convoluted tubules of nephrons; the glucose is consequently excreted in urine [85]. Given that glycosuria is a direct result of SGLT2i, UTI was a major concern as SGLT2i use was approved by FDA for the treatment of T2DM in 2013.  Table 4 shows the results from randomized clinical trials comparing the effects of SGLT2i administration with either placebo or other oral antidiabetic drugs used to increase insulin release such as metformin, dipeptidyl peptidase-4 inhibitor (DPP4i *aka* gliptins), and/or sulfonylureas on the incidence of UTI adverse events. Meta-analyses or pooled data analyses are excluded from Table 4 to avoid data duplication.

Additional observations from SGLT2i clinical trials not included in Table 4 are as follows: (i) A self-controlled case series investigating UTI risk in T2DM patients treated for 4 weeks with SGLT2i (*N* = 2949; women = 80.4%; >50 years of age = 88.7%) reported that women over ≥50 years of age are at the highest risk for UTI (IRR = 1.25) during the first two weeks after initiating SGLT2i therapy (IRR = 1.49) [86]; (ii) a retrospective cohort study of type 2 diabetic elderly women from Canada observed that in comparison to DPP4i treated (*N* = 22,463; 46.1% women; mean age = 74.8 ± 6.7 years), those receiving SGLTi (*N* = 21,444; 41.3% women; mean age = 71.8 ± 5 years) did not experience increased UTI risk (HR = 0.89; *P* = 0.05) and women over >75 years of age showed a modest increase in UTI risk (HR = 1.08) [87]; (iii) similarly in a study of elderly (>65 years old) with T2DM from South Korea, when compared to the new users of DPP4i, the new users of SGLT2i showed a small increase in UTI risk (HR = 1.05; *P* = 0.047) [88]; although in a pooled analysis of outcomes for T2DM patients treated with SGLT2i (empagliflozin, canagliflozin, or dapagliflozin) or placebo, the difference in the UTI risk ratio for men (RR = 1.06) and women (RR = 0.97) was not statistically significant [89]; (iv) a study assessing two large, US-based commercial claims databases showed that SGLT2 inhibitor treatment did not significantly increase the risk of either severe UTI (defined as pyelonephritis or urosepsis requiring hospitalization) in comparison to treatment with active comparators such as DPP4 inhibitor (HR = 0.68) or glucagon-like peptide-1 receptor agonist (HR = 0.78) [90]; (v) a 12-week-long canagliflozin therapy compared with either placebo or sitagliptin was not a significant risk factor of bacteriuria (OR = 1.23; *P* = 0.82) or UTI (aOR = 2.39; *P* = 0.23) [91].

In summary, these observations establish that SGLTi therapy causes a modest increase in the incidence of urinary adverse events such as UTI and bacteriuria, although these urinary adverse events are more common at onset of SGLT2i therapy, are of mild to moderate severity without dissemination to the upper urinary tract, are responsive to antibiotic treatment, and rarely require discontinuation of SGLT2i therapy [91, 92].

While glycosuria enhances growth of uropathogenic bacteria, its effects of bacterial physiology have not been examined. In this regard, we exposed Gram positive GBS to human urine supplemented with 300 mg/dl glucose for 2 h and observed augmentation of virulence characteristics such as adherence to human bladder epithelium, hemolysis, and resistance to antimicrobial peptide LL-37, and a higher urinary burden of glycosuria-exposed GBS in a mouse model of ascending UTI [93]. The effects of glycosuria on UTI pathogenesis have also been studied by experimental induction of ascending UTI in SGLT2i-treated mice [94, 95]. In dapagliflozin- or canagliflozin-treated CBA/J mice, transurethral inoculation with UPEC strain CFT073 or *K. pneumoniae-*KPPR1 significantly increased bladder and urine bacterial burden and resulted in higher bacterial dissemination to spleen at 24 hpi; this was attributed to significant reduction in the levels of proinflammatory cytokines IL-6, IL-1*β*, and TNF*α* and neutrophil myeloperoxidase (MPO) in the urine of SGLT2i-treated mice [94]. Notably, IL-6, IL-1*β*, TNF*α*, or MPO levels were not significantly altered in bladder or kidney tissues of SGLT2-glycosuric mice; urine levels of these analytes in glycosuric mice also returned to non-glycosuric levels by 24 hpi [94]. In addition, in a mouse model of *Candida albicans* ascending UTI on day 5 post-infection, SGLT-2 treated mice showed higher fungal burden in kidneys, which was attributed to persistently increased glycosuria up to 24 h after administration of dapagliflozin and canagliflozin [95]. In contrast, tofogliflozin, which increased glycosuria for 12 h after administration, did not increase renal *Candida* burden [95].

## 9. Sex and Age as UTI Risk Factors in Addition to DM

Stratification of UTI incidence data into men and women subgroups and various age categories within diabetic and nondiabetic cohorts has revealed the influence of these factors on UTI susceptibility. The results from such analyses reveal that both age and female sex increase the risk of UTI independent of DM, although diabetic women are at >3-fold increased risk of ASB and UTI in comparison to men as shown in Table 5 and that the magnitude of UTI risk is different at each age category [66, 76, 77, 96–99]. Moreover, among diabetic individuals, old age and age-associated frailty increase UTI risk by ~2-fold and the need for hospitalization for the treatment of UTI by 1.45-fold [63, 96, 100].

Other interesting observations from subgroup analyses are narrated below:

1. In a majority of studies, when stratified by sex, diabetes increases UTI risk within men and women subgroups by a similar magnitude: (i) UTI RR for T2DM versus no T2DM was 1.49 in men subgroup and 1.53 in women subgroup in a study from the UK [77]; (ii) UTI OR for DM versus no DM was 1.23 in men subgroup and 1.24 in women subgroup in a study from Israel [101]; although (iii) in another study from the UK, UTI OR (T2DM versus no T2DM) was 1.91 in men subgroup, and 1.43 in women subgroup [66]
2. Age increases the risk of UTI by a higher magnitude in the subgroup of diabetic men compared to that in the subgroup of diabetic women: (i) The prevalence of UTI in diabetic women from the US was around 13% across all age categories, while it was positively correlated with increasing age in the subgroup of diabetic men [4]; (ii) a consistent reduction was observed in the fold difference in the UTI incidence rate between diabetic women versus diabetic men; from 3.74-fold at 65-69 years of age, 3.02-fold at 70-74 years, 2.47-fold at 75-79 years, 2.09-fold at 80-84 years, to 1.57-fold at ≥85 years [102]
3. In the youngest age categories (18-24 years for the UK study, 18-39 years for the US study, and 18-50 years for the Spain study), UTI RR for men with T2DM versus men without T2DM was significantly (2–2.2-fold) higher in comparison to UTI risk ratios for women in the same age category [66, 77, 96]
4. Interestingly, when compared with nondiabetic controls, the fold change in UTI risk in those with T2DM consistently decreases with increasing age categories in both men and women which may be attributed to the emergence of aging-associated competing risk factors [66, 77, 96]

Overall, the subgroup analysis of UTI incidence according to sex and age within diabetic and nondiabetic cohorts suggest that female sex and increasing age are positively correlated with the risk of UTI. Although, it must be noted that there are differences in the magnitude of UTI risk in diabetic versus nondiabetic individuals in different age categories and depending on sex. In this regard, mouse experiments examining UTI pathogenesis in male versus female and young versus old subgroups within diabetic and nondiabetic mouse groups will help us decipher mechanistic underpinnings of sex and age as additional risk factors for UTI in diabetic individuals.

## 10. Conclusions

1. The increased risk of UTI in diabetic individuals may be attributed to changes in the host physiology and immune impairment due to hyperglycemia and glycosuria
2. The percent share of various uropathogenic bacterial genera causing UTI in diabetic individuals is similar to that in nondiabetics, although the presence of diabetes increases susceptibility to drug-resistant uropathogens
3. Female sex and advanced age increase the risk of UTI in both diabetic and nondiabetic individuals

## 11. Future Studies

Going forward, the diabetes UTI research should be focused on filling various knowledge gaps with an eye toward developing novel treatments and preventative strategies to mitigate risk of UTI in diabetic patients.

1. In addition to STZ-induced diabetic mice and *db/db* mouse model of DM, other commercially available genetic models such as Akita mouse model of insulin-dependent diabetes due to spontaneous mutation in *insulin 2* gene or NOD (non-obese diabetic) mouse model of autoimmune T1DM may also be adapted for use in UTI research. With the help of these mouse models, future research efforts will improve our understanding of host urinary immune defenses and bacterial virulence factors in the context of diabetes and both complicated (catheter-associated) and uncomplicated UTI
2. In addition to further delineating multifactorial pathogenesis of UPEC, *K. pneumoniae*, *E. faecalis*, and *GBS* in the diabetic urinary tract, future research should also focus on defining the uropathogenesis of *P. mirabilis*, *P. aeruginosa*, and methicillin-resistant *S. aureus*, in which continual emergence of antibiotic resistant strains is a major problem
3. The animal models of diabetes can also be adapted for gaining cellular and molecular insights into the role of age and sex for both complicated and uncomplicated UTI caused by different uropathogens
4. Ascending UTI induced in nondiabetic mice treated with SGLT2 inhibitor or vehicle control may be used to differentiate the effects of glycosuria from the effects of diabetic urinary microenvironment on the pathogenesis

## Figures and Tables

**Table 1:** Parameters and results for experimental induction of ascending UTI in diabetic mice.

Pathogen^a^ [strain]	Mouse strain	Method of DM induction^b^	Average glucose in blood (HG^c^) and urine (GU^d^) and mouse weight (wt^e^)	Main findings	Ref
GBS [COH1]	CD1	4 IP doses of 80 mg/kg STZ to 8 wk old mice	HG: 393 (STZ); 142 (Veh) GU: 478 (STZ); 96.5 (Veh) Wt: 34.6 (STZ); 40.6 (Veh)	Increased GBS burden in bladder, kidneys of STZ-mice may be due to detrimental effects of high cathelicidin	[12]
UPEC [UTI89]	*db/db*	Genetic model, STZ not administered	HG: 607 ± 27.3 (*db/db*), 201 ± 13.1 (C) GU: 440 ± 166 (*db/db*), ND (C) Wt: 33.83 ± 0.75 (*db/db*), 19.5 ± 0.5 (C)	Significantly higher UPEC burden in bladder, urine of *db/db* mice due to reduced signaling through renal IR—AKT axis	[16]
UPEC [UTI89]	TALLYHO	Genetic model, STZ not administered	HG: 183 ± 32 (Tallyho), 130 ± 8.5 (C) GU: NR (Tallyho), NR (C) Wt: 24.33 ± 0.81 (Tallyho), 16.17 ± 0.4 (C)	Significantly higher UPEC burden in urine of TALLYHO mice due to reduced signaling through renal IR—AKT axis	[16]
UPEC [53498]	C57BL/6 J	1 or 2 IP doses of 150 mg/kg STZ in 8 wk old mice	HG: 562 ± 32.1 (STZ); 111 ± 8.5 (Veh) GU: NR Wt: 18.5 ± 1.07 (STZ); 22.4 ± 1.13 (Veh)	Increased accumulation of AGEs on uroepithelial cells increases UPEC adherence	[11]
UPEC [53498]	C57BL/6 J	2 IP doses of 150 mg/kg STZ in 8 wk old mice	HG: 524.2 ± 9.7 (STZ); 106.4 ± 3.5 (Veh) GU: NR Wt: 21.6 ± 0.63 (STZ); 22.4 ± 0.75 (Veh)	Reduced transcript levels for IL-6, CXCL1, CXCL2, and CCL2 and reduced neutrophil recruitment in diabetic mice	[10]
UPEC [UTI89], *Kp* [TOP52], *Ef* [0852]	C57BL/6 C3H/HeN C3H/HeJ	2 or 3 IP doses of 200 mg/kg STZ in 4-5 wk old mice	HG: >250 mg/dl selected for infection GU: >200 (STZ), ND (Veh) Wt: NR	Induction of interstitial pyelonephritis marked by renal neutrophilia and inflammation	[9]

Pathogen^a^ names are shown: GBS, group B *Streptococcus* (*Streptococcus agalactiae*); UPEC: uropathogenic *Escherichia coli*; *Kp*: *Klebsiella pneumoniae*; and *Ef*: *Enterococcus faecalis*. STZ treatment regimen^b^: amount of streptozotocin, frequency of administration, and mouse age at which STZ treatment was administered via IP (intraperitoneal) route are shown. Control mice were injected equivolume 0.1 M sodium citrate (pH 4.5) at the same frequency via IP route. Mouse characteristics show average blood glucose (HG^c^, hyperglycemia in mg/dL), average urine glucose (GU^d^, glycosuria in mg/dl), and mouse weight (Wt^e^) in Gram in diabetic (STZ, *db/db*, or Tallyho) or control (Veh, C) mice. Where available, data are shown as average ± standard deviation. NR indicates data not reported.

**Table 2:** Epidemiological studies examining incidence and prevalence of community-acquired UTI in diabetic individuals.

Country [year] study type	DM type	Mean age (years)^b^		Sample size [% women]		Measures of association^a^	Ref
		DM	Non-DM	DM	Non-DM		
Canada [2021]	T1DM, T2DM	74^b^ [63–82]	72^b^ [53–84]	41,934 [53.3]	108,565 [48.4]	PR = 1.16	[8]
Spain [2020]	T2DM	77.1	66.4	216,741 [59.1]	633,535 [59.5]	IRR = 4.36	[96]
Pakistan [2020]	T2DM	46 ± 11	51 ± 13	512 [56.3]	562 [55.5]	IRR^calc^ = 2.14	[29]
Portugal [2020]	T2DM	70.9	NI	7,347 [48.9]	NI	IR = 16.2%	[97]
China [2020] P	NR	NR	NR	1804 [NR]	22,181 [NR]	OR = 1.59; *P* = 0.014	[103]
Iran [2019] CS	T2DM	62.3 ± 14.4	NI	700 [53.4]	NI	IR = 11.3%	[104]
S. Korea [2018]	T2DM	58.2 ± 13.3	58.2 ± 13.3	66,426 [50.4]	132,852 [50.4]	IRR = 1.83	[105]
UK [2018]	T2DM	67.6	NR	96,630 [44.7]	191,822 [44.8]	IRR = 1.53	[6]
	T1DM	56.5	NR	5,863 [41.5]	11,696 [41.5]	IRR = 1.81	
China [2018]	T2DM	59.3 ± 14.1	NI	3,652 [38.7]	NI	IR = 11.2%	[34]
US [2017]	T2DM	60.2 ± 12.8	60.2 ± 12.7	39,295 [47.8]	39,295 [47.8]	IRR = 1.25; *P* < 0.001	[106]
US [2016]	T1DM	50.7 ± 7.2	NI	572 [100]	NI	IR = 15%	[75]
UK [2016]	T2DM	67 [57–76]	46 [33–61]	34,278 [43.8]	613,052 [51.3]	OR = 1.59; *P* < 0.001	[74]
Australia [2016] P	T2DM	Overall = 37.1 ± 15.3		396 [48]	2391 [48]	aRR = 3.6	[65]
Denmark[2016] MC	T2DM	65.6 ± 13.6	65.7 ± 13.6	155,158 [45]	774,017 [45]	aRR = 1.41	[107]
Germany [2015]	T2DM	72.8 ± 12.3	NI	456,586	NI	IR^DM^ = 87.3/10^3^PY	[76]
US [2014]^c^	T2DM	56 ± 13.4	56 ± 13.4	89,790 [49.3]	89,790 [49.3]	IRR^calc^ = 1.68 OR^calc^ = 1.75	[66]
US [2014]	T2DM	60.6^d^	NI	73,151 [47.9]	NI	PR = 8.2%	[4]
US [2013]	T2DM	63.1 ± 11.7	56.9 ± 15.5	2,671 [60.3]	8,907 [62.3]	OR = 1.54; *P* < 0.0001	[98]
UK [2014]	T2DM	71 [65–77]	NI	218,805	NI	IR = 99.6/10^3^PY	[102]
Saudi Arabia [2013]	T2DM	51.9 ± 15.9	NI	1000 [53.1]	NI	PR = 25.3%	[108]
UK [2012]	T2DM	62.6 ± 13.5	62.6 ± 13.7	135,920 [46]	135,920 [46.1]	IRR = 1.53	[77]
Brazil [2012]	NR	Overall = 71.9 ± 6.26		119 [100]	479 [100]	OR = 1.77; *P* = 0.031	[61]
Sweden [2010] R	T2DM	57.4 ± 10.9	NI	6016 [50.7]	NI	IR = 59.5/10^3^ PY	[100]
Netherlands [2010]	T1DM	63 ± 17	51 ± 16	50	6618	aOR = 1.9	[71]
	T2DM	66 ± 14		290		aOR = 2.0; *P* < 0.001	

Measures of association^a^ shows the incidence rate (IR) or prevalence rate (PR) where nondiabetic subjects were not included or incidence rate ratio (IRR) or odds ratio (OR). The superscript suffix ^calc^ refers to OR/IRR calculated using published data. Mean age (years)^b^ ± standard deviation are shown. In some cases, where mean age is not provided median age [interquartile range] are shown. US [2014]^c^; In this study, occurrence of UTI in 1 year follow-up period in all subjects was used to calculate prevalence rate. In contrast, subjects without prior history of UTI (*N* = 82,239) were grouped as incidence cohort, and the occurrence of UTI in incidence cohort during 1 year follow-up period was used to calculate incidence ratio. ^d^Calculated average age based on the provided information. NR∗ refers to data not reported; NI^&^ to not included.

**Table 3:** Studies examining incidence and prevalence of healthcare-associated UTI in diabetic individuals.

Country [year]	Comorbidity or reason for hospitalization/homecare	Sample size	% F^a^	%DM^b^	Age^c^	Measures of association^d^	Ref
Taiwan [2021]	Diabetic chronic kidney disease	79,887	30.3	100	59.6 ± 14.0	IR = 39.8/10^3^ PY	[63]
US [2021]	Total knee arthroplasty	189,327	NR	NR	NR	OR = 1.34; *P* < 0.0001	[109]
Thailand [2020]	Ischemic stroke	370,527	46.6	20.4	>65	OR = 1.34; *P* < 0.001	[110]
	Hemorrhagic stroke	173,236	40.3	9.8	>61	OR = 1.25; *P* < 0.099	
	Undetermined stroke	65,127	NR	18.1	NR	OR = 1.54; *P* = 0.013	
US [2019]	Suprapubic catheterization post pelvic organ prolapse surgery	254	100	12	65.5^g^	OR = 2.80; *P* = 0.01	[111]
Taiwan [2019]	Bed-bound elderly on homecare	598	60.5	46.5	81.9 ± 11.3	OR = 1.46; *P* = 0.024	[112]
US [2018]	Traumatic thoracic vertebral fracture repair	1088	31.7	50	61	OR^calc^ = 1.7; *P* = 0.036	[113]
Canada [2018]	Subarachnoid hemorrhage	419	63.7	9.3	58 [48–67]	HR = 1.92	[114]
India [2018]	Renal transplant	210	NR	NR	NR	60.71% UTI+ had new- onset post-transplant DM	[115]
Japan [2018]	Cerebral infarction with indwelling urinary catheter	27,548	52	23	76 ± 12	OR = 1.43^e^; *P* < 0.001 OR = 0.91^f^; *P* = 0.24	[116]
Poland [2018]	Radical cystectomy	134	23	19.4	65.9	OR = 3.75; *P* = 0.026	[117]
Spain [2017]	Total hip/knee arthroplasty	74,835	62.7	50	71.5	OR^alc^ = 1.31	[118]
US [2017]	Endoscopic sinus surgery	644	50	13.2	NR	OR^alc^ = 6.78; *P* =0.03	[119]
Australia [2017]	Anterior cervical discectomy and fusion (ACDF)	3725	49.9	11.8	NR	OR^alc^ = 2.2	[120]
China [2016]	Hospitalized diabetic elderly	817	49.2	100	≥60	3.2%	[121]
US [2016]	Radial cystectomy to treat bladder cancer	3187	18.2	19.6	70 [62–77]	9.7% developed UTI OR = 0.96; *NS*	[122]
US [2016]	Radial cystectomy to treat bladder cancer	1248	16.8	16.9	69 [61–76]	10% developed UTI OR = 2.27; *P* < 0.001	[123]
Taiwan [2015]	Stroke	221,254	39.5	4.5	64	OR = 1.66	[124]
Yemen [2015]	Renal transplant-one year follow up	150	38	46	35.1	RR = 2.43; *P* = 0.014	[125]
US [2016]	Head and neck cancer surgery	31,075	58	13	61.6^g^	OR = 1.048^h^; *NS*	[126]
US [2015]	Cardiac surgery, urinary catheter	4,883	33.4	31.3	NR	OR = 2.04; *P* = 0.013	[36]
US [2014]	Emergency abdominal surgeries	53,879	55.2	20.2	76.2^g^	OR = 1.32; *P* < 0.001	[127]
US [2014]	Total elbow arthroplasty	3,184	67.5	15.3	59.7	OR = 2.24; *P* < 0.001	[128]
US [2014]	Elective lumbar fusion	15480	55.9	15.7	NR	RR = 1.6 − IDDM; *P* = 0.011 RR = 1.0 − NIDDM; *NS*	[129]
Sweden [2014]	Ultrasound-guided prostate biopsy	51,321	0	8.6	NR	OR = 1.32	[130]
Turkey [2013]	Hospitalized for various reasons	930	49.8	50	62.7-DM 54.6-no DM	OR^calc^ = 1.4^g^	[39]
Spain [2012]	Solid organ transplant	4,388	33.2	18.8	50 ± 14.5	OR = 1.01^i^; *NS* OR = 1.02^j^; *P* = 0.037	[131]
China [2011]	Acute ischemic stroke	12,907	38.2	27	67 [56–75]	OR^calc^ = 1.61; *P* < 0.0001	[132]
Sweden [2010]	Radial cystectomy, urinary catheter	452	23	88.9	30-80	RR = 2.1	[133]
US [2010]	Non-cardiac surgery	3112	53.8	20	56.5 ± 16	OR^calc^ = 3.28	[134]

%F^a^ refers to percentage of female subjects and %DM^b^ to percentage of diabetic subjects. Age^c^ refers to the average age in years ± standard deviation or [interquartile range] is shown. NR refers to not reported. Measures of association^d^: Incidence rate (IR) (shown as IR per 1000 persons per year), hazard ratio (HR), incidence rate ratio (IRR), or odds ratio (OR) for UTI in diabetic individuals is shown. Where available, *P* values are shown; *NS* refers to not significant. ^e,f^Odds ratio calculated by multivariate regression for diabetics treated with insulin^e^ or not^f^. ^g^ Calculated average age based on the provided information. ^h^Odds ratio adjusted for race and sex was NOT statistically significant (*P* = 0.697). ^i,j^Odds ratio for UTI in DM versus no DM in the kidney and kidney-pancreas transplant patients^i^ or in the liver, heart, and lung patients^j^.

**Table 4:** UTI adverse events as reported in SGLT2i clinical trials.

Study characteristics^a^	% UTI incidence [sample size] in various treatment cohorts^b^						Ref
	Placebo	Oral antidiabetic (OAD) treatment cohorts					
Various oral antidiabetic drugs; 154 weeks	Not included	SGLT2i 11.9 [11,364]	DPP4i 11.9 [9035]	Biguanide 10.5 [10,359]			[135]
Ipragliflozin (Ip) + sitagliptin (S); 24 weeks	Ip0 + S 1.4 [70]	Ip50 1.4 [73]					[136]
Bexagliflozin (B); 24 weeks All subjects had CKD	B0 3.2% [155]	B20 7% [157]					[137]
Dapagliflozin (D), safagliptin (Sf), or glimepiride (GL) + metformin (M); 52 weeks	Not included	D10 + M 7.7 [313]	D10 + Sf5 + M 4.2 [312]	GL1-6 + M 3.8 [312]			[138]
Dapagliflozin (D) + insulin (in); 24 weeks	D0 + In 5.3 [133]	D10 + In 3.6 [139]					[139]
Tofogliflozin (T); 52 weeks	T0 1.5% [68]	T20 2.1% [140]					[140]
Ertugliflozin (E) ± sitaglitpin (S);26 weeks	Not included	E5 5.2% [250]	E15 5.6% [248]	S100 3.2% [247]	E5 + S100 3.3% [243]	E15 + S100 3.7% [244]	[141]
SGLT2i or DDP4i	Not included	SGLT2i 3.6 [1977]	DPP4i 4.9 [1964]				[142]
Ertugliflozin (E); 52 weeks	E0 8.5% [153]	E5 7.1% [156]	E15 3.9% [152]				[143]
Dapagliflozin (D) + insulin	D0 + In 5 [60]	D5/D10 + In 1.6 [123]					[144]
Dapagliflozin (D) + metformin (M); 24 weeks	D0 + M 4.8% [145]	D5 + M 4.1% [147]	D10 + M 6.6% [152]				[145]
Dapagliflozin (D) + saxagliptin (Sx) + metformin (M); 24 weeks	D0 + Sx5 + M 6.3 [160]	D10 + Sx5 + M 5 [160]					[146]
Canagliflozin (C); 18 weeks	C0 4.9% [226]	C100 3.1% [223]	C300 2.6% [227]				[147]
Dapagliflozin (D); 24 weeks	D0 3.7% [1393]	D2.5 3.6% [814]	D5 5.7% [1145]	D10 4.3% [1193]			[148]
Dapagliflozin (D); 24 weeks	D0 3% [132]	D5 3.9% [128]	D10 3.8% [133]				[149]
Dapagliflozin (D) + metformin (M); 102 weeks	D0 + M 4.4% [91]	D10 + M 3.3% [91]					[150]
Canagliflozin (C); 52 weeks	C0 5.6% [90]	C100 5.6% [90]	C300 7.9% [89]				[151]
Empagliflozin (E) + metformin (M); 12 weeks	E0 2.8% [71]	E1 + M 2.8% [71]	E5 + M 2.8% [71]	E10 + M 4.2% [71]	E25 + M 5.7% [70]	E50 + M 4.3% [70]	[152]
Empagliflozin (E); 12 weeks	E0 1.2% [82]	E5 2.5% [81]	E10 1.2% [81]	E25 1.2% [82]			[153]
Ipragliflozin (Ip), metformin (M); 12 weeks	Ip0 8.7% [69]	Ip12.5 5.7% [70]	Ip50 13.4% [67]	Ip150 1.5% [68]	Ip300 10.3% [68]	M 7.2 [69]	[154]
Ipragliflozin (Ip); 12 weeks	Ip0 6.1% [66]	Ip12.5 1.4% [69]	Ip50 2.9% [68]	Ip150 6.0% 67]	Ip300 6.9% [72]		[155]
Dapagliflozin (D); 12 weeks	D0 1.9% [54]	D1 1.7% [59]	D2.5 0% [56]	D5 1.7% [58]	D10 3.8% [52]		[156]
Canagliflozin (C); 26 weeks	C0 4.2% [192]	C100 7.2% [195]	C300 5.1% [197]				[157]
Saxagliptin (Sx) + (insulin (in) ± metformin (M)); 52 weeks	Sx0 + In+M 6% [151]	Sx5 + In+M 5.9% [304]					[158]
Dapagliflozin (D) + metformin (M); 24 weeks	D0 + M 2.2% [91]	D10 + M 6.6% [91]					[159]
Dapagliflozin (D) or glipizide (GL) + metformin (M); 52 weeks	Not included	D2.5 + M 7.4% [406]	GL5 + M 4.2% [408]				[160]
Dapagliflozin (D); 24 weeks	D0 4% [75]	D2.5 6.1% [132]	D5 11.6% [166]	D10 8.1% [185]			[161]

Study characteristics^a^: Specific SGLTi and comparators used in the study are indicated. In studies where metformin was a comparator, its dose was >1500 mg/day. % UTI incidence (sample size) in various treatment cohorts^b^: Specific placebo or oral antidiabetics are shown followed by the amount of drug administered (in mg) every day.

**Table 5:** Studies examining sex as a risk factor of UTI in diabetic individuals^a^.

Study [year]	Sample size	%F^b^	Measures of association^c^	Ref
Ethiopia [2019]	239	60.2	OR = 6.55	[32]
China [2018]	3264	43.3	OR = 10.6; P < 0.001	[34]
China [2016]^d^	817	49.2	OR^calc^ = 4.4; P = 0.004	[121]
UK [2014]^e^	218,805	49.4	IRR^calc^ = 2.9; P < 0.001	[102]
US [2013]^f^	11,578	61.8	OR = 4.2; P < 0.0001	[98]
Saudi Arabia [2013]	1000	53.1	RR = 6.1; P < 0.001	[108]
Sweden [2010]	6,016	50.7	RR = 3.4	[100]

^a^All studies included the cohorts of only diabetic individuals. US [2013]^f^ included both diabetic (23.1%) and nondiabetic cohorts. %F^b^ refers to percentage of female subjects. Measures of association^c^: odds ratio (OR) or relative risk (RR) for UTI in diabetic women vs UTI in diabetic men. China [2016]^d^: All subjects were elderly (>60 years of age) diabetic individuals hospitalized for various reasons. UK [2016]^e^: All subjects were elderly (>65 years of age) diabetic individuals.

## Data Availability

Data will be made available on request.
